# Long-Term Effect of Simulation-Based Education on Knowledge Retention in Initial Neonatal Care Among Nurse-Midwives in Kilimanjaro, Tanzania: A Quasi-Experimental Study

**DOI:** 10.3390/nursrep16100365

**Published:** 2026-10-15

**Authors:** Rosemary Michael Malya, Michael Johnson Mahande, Kristin Hjorthaug Urstad, Bodil Bø, Jane Rogathi

**Affiliations:** 1 Department of Midwifery, School of Nursing, KCMC University, Moshi P.O. Box 2240, Tanzania; 2 Department of Obstetrics and Gynaecology, Kilimanjaro Christian Medical Centre Hospital, Moshi P.O. Box 3010, Tanzania; 3 Department of Epidemiology & Biostatistics, KCMC University, Moshi P.O. Box 2240, Tanzania; michael.mahande@kcmcu.ac.tz; 4 Faculty of Health Sciences, University of Stavanger, 4021 Stavanger, Norway; kristin.h.urstad@uis.no (K.H.U.); bodil.bo@uis.no (B.B.); 5 Department of Community, School of Nursing, KCMC University, Moshi P.O. Box 2240, Tanzania; jane.rogathi@kcmcu.ac.tz

**Keywords:** simulation-based education, knowledge retention, initial neonatal care, nurses-midwives, Tanzania

## Abstract

**Background:** Although simulation-based education is reported to improve nursing and midwifery students’ clinical competencies, evidence regarding knowledge retention remains limited, particularly in low-resource settings. This study evaluated two-year knowledge retention following simulation-based education in initial neonatal care among nurse-midwives in Tanzania. **Methods:** This is a two-year follow-up of **a** quasi-experimental study conducted in June 2025 among 84 nurse-midwives: 43:41 to the intervention and control groups. Participants were among the 90 second-year diploma nursing and midwifery students from a quasi-experimental reference study conducted between March and June 2023, with 45 students in the intervention group and 45 in the control group. Both study groups received lectures, while the intervention group received additional simulation-based education on initial neonatal care. Data were collected using a structured, electronic questionnaire—the same questionnaire employed in the reference study. Data were analyzed using descriptive statistics, paired *t*-test, McNemar’s exact test, and Generalized Linear Mixed Models. **Results:** After two years of simulation-based education on initial neonatal care, knowledge declined in both groups but remained higher in the intervention group (16.3 vs. 12.7), with higher knowledge retention (7.2 vs. 4.2 points). The adjusted difference-in-differences analysis showed no significant long-term effect of simulation on knowledge retention (aDiD = 0.06; 95% CI: −1.47 to 1.60; *p* = 0.935). **Conclusions:** The intervention group had higher observed knowledge scores at follow-up, but the study did not demonstrate a statistically significant differential long-term effect of the single SBE intervention. **Implication:** Integrating simulation-based education into nursing curriculum and professional training may enhance knowledge retention.

## 1. Introduction

Simulation-based education (SBE) is widely recognized as an effective educational pedagogy in nursing and midwifery education, particularly among institutions in developed countries [1,2]. The implementation of this pedagogical approach in these contexts is related to SBE’s ability to replicate real-world clinical scenarios, enabling students to apply theoretical knowledge and develop practical skills within a safe and controlled learning environment [3,4]. Nursing education aims to prepare students to translate theoretical knowledge across a broad range of clinical situations, from routine patient care to emergency cases [5]. This is particularly important because high-quality nursing and midwifery practice relies on both knowledge and hands-on clinical skills.

The Tanzanian professional nurse-midwives practicing in various fields of nursing and midwifery, including maternal and neonatal care, use the knowledge and skills they have acquired from the Ordinary Diploma Nursing and Midwifery education [6]. This suggests that pre-service education is the main source of competence development for students and aspiring skilled professional nurse-midwives [6]. The national diploma curriculum emphasizes competency-based learning to prepare graduates with clinical competencies [7]. However, the traditional lecture methods of teaching and skills demonstrations in the skills laboratory are mainly implemented in the pre-service nursing education to prepare students for clinical practice [8]. While such teaching strategies provide theoretical foundations, they may limit students’ opportunities for hands-on exposure to real-world skills [9]. The curriculum for diploma nursing and midwifery was reviewed in 2017, and simulation-based education was listed as a teaching method [10].

Strengthening healthcare providers’ (HCPs), including nurse-midwives’, competence in initial neonatal care (INC) is important in Tanzania, where neonatal mortality remains a major health challenge [11,12]. The Tanzania neonatal mortality rate was 24 deaths per 1000 live births in 2022, with birth asphyxia being the leading cause of deaths [13]. The first hour after birth is critical for neonatal survival, requiring timely assessment and evidence-based initial neonatal care (INC) focusing on the World Health Organization and Tanzania’s national neonatal care guidelines [14,15,16]. INC includes assessment of breathing, thermal protection through drying and skin-to-skin contact, early initiation of breastfeeding, delayed cord clamping, and resuscitation when indicated. The implementation of these interventions by skilled and knowledgeable healthcare providers (HCPs) might prevent early complications, while reducing neonatal mortality.

Although Tanzania aimed to achieve 90% coverage of deliveries attended by skilled HCPs capable of providing INC by 2025 [17], the coverage has increased from 64% (2016–2021) to 84.76% in 2022 [18,19]. However, the improvements in the quality of care have not matched this increase [17]. The 2018 Lancet Global Health Commission recommended strengthening pre-service healthcare education in low-resource settings through active learning, early clinical exposure, and problem-based learning to improve HCPs’ competence and the quality of care [20]. However, few countries in these contexts participated in subsequent discussions on how to reform and improve the quality of their healthcare systems [21]. A subsequent report on High Quality Health Systems 1 year On, further emphasized competency-based HCP training as essential for improving healthcare quality [21].

Simulation-based education (SBE) enhances nursing students’ knowledge, clinical skills, and long-term retention of learning [22,23]. SBE also improves critical thinking, communication and teamwork, which are essential for professional practice [24,25]. While a few studies examined SBE in nursing and midwifery education in Tanzania [26,27,28], evidence on its long-term effect on INC knowledge retention beyond one year remains limited. Most SBE studies evaluate outcomes immediately after training or within a few weeks or months [29,30]. Since SBE is still a new pedagogy in Tanzanian nursing and midwifery education, this study aimed to evaluate the long-term effect of SBE on knowledge retention in INC that was tested in the previous reference study [31].

**Hypothesis 1:** 
*Nurse-midwives receiving simulation-based education have higher long-term knowledge retention of initial neonatal care than those receiving lecture teaching methods.*

## 2. Materials and Methods

### 2.1. Study Design

This is a long-term, two-year follow-up of a quasi-experimental study that examined the effect of SBE on knowledge in the reference study [31]. The study evaluated the effect of SBE on long-term knowledge retention in initial neonatal care (INC) among nurse-midwives in Kilimanjaro, Tanzania.

### 2.2. Study Population and Setting

The study included 84 nurse-midwives who participated as second-year diploma in nursing and midwifery students in the reference quasi-experimental study conducted between March and June 2023 at two nursing schools: one located in an urban area and the other in a rural area in Kilimanjaro, Tanzania [31]. The reference study was conducted in two of the six schools offering diploma-level nursing and midwifery education in Kilimanjaro, Tanzania. Two schools were purposively selected: one located in an urban area was designated as the urban school and served as the intervention group, while the other, located in a rural area, was designated as the rural school and served as the control group.

In the reference study [31], 90 students were purposively recruited: 45 from an urban school as the intervention group, and 45 from a rural school as a control group. Second-year diploma in nursing and midwifery students were selected because the Tanzanian Ordinary Diploma in Nursing and Midwifery curriculum introduces maternal and neonatal care, including essential newborn care and neonatal resuscitation, referred to as “Initial Neonatal Care (INC)” in the current study, during the second academic year. The Diploma in Nursing and Midwifery program takes three years of training [32,33], and follows similar standards across all institutions, regardless of geographical location, including comparable curricula, enrolment capacity, and human resources. Therefore, compared with the other schools, the rural school had comparable characteristics to those of the urban school. Thus, before initiation of the reference study, students from both groups had received 2 h INC lecture teaching conducted as routine sessions in classrooms by nurse tutors before students’ clinical practice placements. Of the 90-reference cohort, 84 nurse-midwives were recruited: 43 in the intervention and 41 in the control group, with 6.7% lost to follow-up. After graduation, the nurse-midwives were dispersed across different geographical locations and had left their mobile phone numbers with the researcher. At the time of follow-up, some participants could no longer be reached by phone, and their colleagues were unable to provide updated contact information.

### 2.3. Sample Size

Sample size calculation was based on the pre-test—post-intervention reference study and did not specifically account for the two-year DiD analysis [31]. In the reference study, sample size was designed to determine how many participants needed to be recruited in the study. However, the current study was designed as a cohort follow-up of all 90 eligible students who participated in the reference study during the two-year follow-up period. Therefore, 84 nurse-midwives included in the current study were determined by the number of 90 eligible graduates available during the follow-up period, rather than by a statistical sample-size calculation.

### 2.4. Participants Allocation to the Intervention and Control Groups

Participants were allocated to the intervention and control groups at the school level. In the reference study [31], two nursing schools were purposively selected from six schools offering diploma-level nursing and midwifery programs in Kilimanjaro. Of the six schools, three are located in urban areas, while the other three are located in rural areas. One of the three urban schools was purposively selected and designated as the intervention group because the school was near the university, which had infrastructure for SBE, i.e., a simulation skills laboratory. From the three rural schools, one was selected and designated as the control group because it had characteristics comparable to those of the urban school, including student enrolment capacity, human resources, and curriculum. The selected rural school was chosen to ensure minimal interaction and prevent cross-contamination of information between the study groups, as students from the urban and the selected rural school attended different clinical placement sites during their clinical rotations. In contrast, the other rural schools shared similar clinical placement sites with the urban school for skills practice.

Notably, although the two schools were located in different settings (urban and rural), neither had a skills laboratory with infrastructure to support simulation-based education (SBE). The schools were named “urban and rural nursing schools” for study confidentiality.

### 2.5. Simulation-Based Education (SBE) Implementation

The SBE described in this study was conducted in June 2023 among 45 second-year diploma nursing and midwifery students from a reference quasi-experimental study examining the effectiveness of simulation-based education on initial neonatal care (INC), and it is well described in the reference study [31]. Among the 90 students, 45 in the intervention and 45 in the control group who participated in the reference study, 45 students from the intervention group participated in the SBE in INC. However, before SBE, both study groups received a two-hour standard lecture on initial neonatal care (INC), while the intervention group received additional simulation-based education. SBE was delivered according to the National League for Nursing (NLN) Jeffries Simulation Theory [4]. The students simulated two different INC scenarios: (1) INC for a normal, healthy-breathing neonate (routine essential newborn care), and (2) INC for neonates with difficulty breathing at birth (neonatal resuscitation) using a low fidelity-Baby NeoNatalie simulator, and a reusable self-inflating Ambu-bag and mask size −1. The equipment were manufactured by Laerdal Global Health, Stavanger, Norway https://www.laerdalglobalhealth.com/. The first and second scenarios were simulated in 8 and 5 min, while the debriefing sessions lasted for 50 min. SBE sessions were held each day in the simulation skills laboratory located at the university, which is near to the urban school. Three students participated actively in scenario simulation as nurse-midwives, while two students were assigned roles to observe initial neonatal care (INC) practices and communication within the team. The SBE was facilitated by the researcher. Each simulation session incorporated a structured pre-briefing, scenario experience, and debriefing sessions based on Gibbs’ Reflective Cycle [34]. Participants’ roles were swapped and repeated to optimize active participation and learning. Both lecture teaching and SBE were facilitated by the researcher. No additional SBE was conducted during the two-year follow-up.

### 2.6. Study Variables and Measurement

The primary outcome variable was knowledge retention, determined by comparing change in knowledge scores between the immediate post-intervention assessment and the two-year follow-up. A secondary outcome variable was the intervention effect estimated using a difference-in-differences (DiD) approach [35]. Knowledge was assessed using the same structured questionnaire administered at pre- and post-intervention in the reference study, enabling comparison of knowledge retention between the intervention and control groups.

### 2.7. Validity and Reliability

The questionnaire contained 22 items of INC assessing knowledge of essential newborn care and neonatal resuscitation based on the Tanzanian nursing and midwifery curriculum, national neonatal care guidelines, and World Health Organisation recommendations [15,16,36]. The questionnaire was previously reviewed by nursing educators, neonatal specialists, and clinical midwives, and pilot-tested among diploma nursing and midwifery students from a different school before its use in the reference study [31]. Reliability was assessed using Cronbach’s alpha, and the results for knowledge were 0.7497. All these steps were undertaken to enhance the credibility, reliability and methodological rigor of the study findings. No modifications were made to the questionnaire during the two-year follow-up period. Each correct response was scored as “1” and each non-retained response as “0”, producing a total score ranging from 0 to 22, with higher scores indicating higher knowledge.

### 2.8. Data Collection

Data was collected from 1 to 30 June 2025 using a structured, electronic questionnaire, filled by the researcher and a research assistant to minimize the risk of respondent bias. However, in the reference study, data were collected using a hard copy, self-administered questionnaire supervised by the researcher because the students were still in the training institution. The same knowledge questions employed in the reference study were employed in the current study [31]. This approach ensured methodological consistency with the reference study and minimized respondent bias [37]. Participants were contacted by mobile phone using contact information collected during the reference study because graduates were geographically dispersed following completion of their training. Electronic written informed consent was obtained to participate in the study. The participants’ unique identification (ID) numbers assigned during the reference study were reserved by the researcher and used in the follow-up study to enable linkage between post-intervention and follow-up data while preserving anonymity. Of the original cohort of 90 participants, six were lost to follow-up, resulting in a retention rate of 93.3%.

### 2.9. Ethical Approval

The study was approved by the KCMC University ethics (ID: 2617) on 7 February 2025 and the Tanzania National Health Research Ethics Committee (NaTHREC) with (ID: 4361) on 3 July 2024, each of the approval lasting for one year. Electronic and written informed consent was obtained from each participant before data collection. The study follows the Declaration of Helsinki ethical principles of conducting research in human subjects [38].

### 2.10. Analysis

Data were analyzed using STATA version 17 (Stata Corp, College Station, TX, USA). Data were summarized using descriptive statistics and inferential statistics; a paired *t*-test for continuous variables and McNemar’s exact test to account for the paired nature of the observations. The long-term effect of SBE on INC knowledge retention was estimated using a Difference-in-Differences (DiD) analysis performed with a Generalized Linear Mixed Model (GLMM). A Gaussian distribution with an identity link was selected because the knowledge score, ranging from 0 to 22, demonstrated characteristics supporting its treatment as an approximately continuous outcome. The score had a relatively large number of distinct values across its observed range and an approximately symmetric distribution, with closely aligned mean and median values; there was also no substantial evidence of floor or ceiling effects.

A difference-in-differences (DiD) approach was subsequently applied to estimate the intervention effect by comparing changes in knowledge scores from the post-intervention assessment to the two-year follow-up between the intervention and control groups. The DiD approach was selected because it accounts for baseline differences between the groups and estimates whether the intervention group experienced a greater change in the outcome over time relative to the control group while accounting for common temporal changes affecting both groups. The model included study phase, study group, and their interaction, with the interaction term representing the DiD estimator and quantifying the differential change in outcomes between the two groups. Because the outcome was modeled as a continuous measure, intervention effects were reported as regression coefficients (β) with corresponding 95% confidence intervals (CIs). A positive β indicated a greater increase or smaller decline in the outcome in the intervention group relative to the control group, whereas a negative β indicated a smaller increase or greater decline in the intervention group relative to the control group. Age, education level, marital status, employment status, and gender were included in the descriptive analysis. Gender was the only fixed-effect covariate included in the adjusted model because it was the only measured post-intervention characteristic that showed evidence of imbalance between the intervention and control groups (*p* < 0.05). The model also accounted for the random effect of study participants to account for the correlation arising from repeated measurements within participants.

Given the small sample size of 84 participants (43 in the intervention group and 41 in the control group), convergence difficulties were encountered when fitting models with multiple covariates. To minimize the risk of over-parameterization, unstable parameter estimates, and reduced precision, the GLMM was therefore specified parsimoniously, with adjustment for gender as the only covariate. Statistical significance was assessed using two-sided tests at the 5% significance level.

Of the 90 students enrolled in the reference study [31], 84 (93.3%) completed the two-year follow-up assessment. The follow-up rate was 95.6% (43/45) in the intervention group and 91.1% (41/45) in the control group. Total follow-up loss was 6.7% (n = 6) out of 90 participants over the two-year follow-up period. The 6.7% non-response rate is below the 10% non-response rate that was adjusted for during sample size calculation in the reference study [31]. Furthermore, it ensures that the baseline equivalence established between the two groups was preserved over time, validating that the integrity of the comparative analysis was not compromised by attrition bias [39,40].

**Figure 1:**
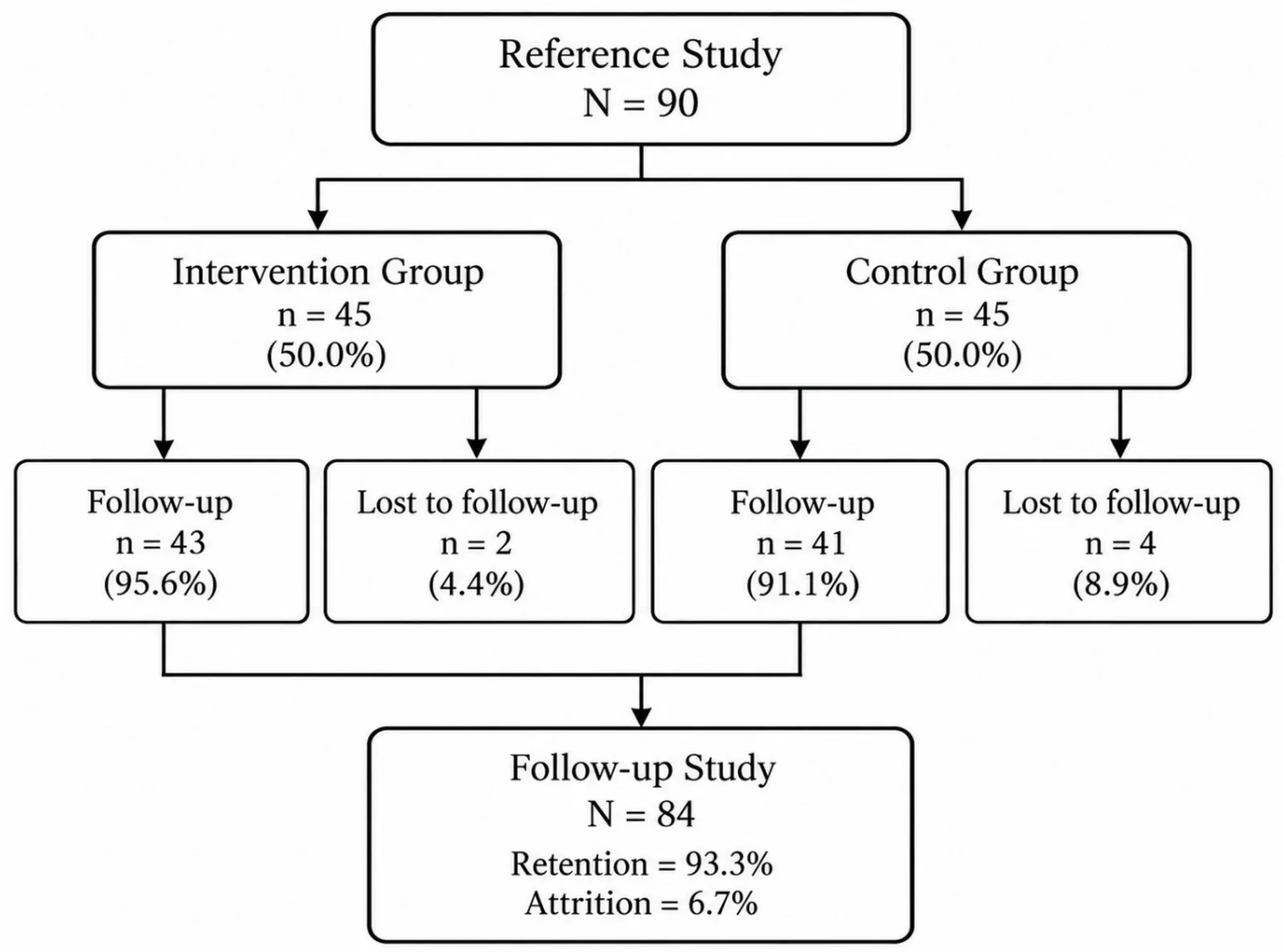
Participant flow from the reference study to the two-year follow-up study.

Figure 1 shows that, of the 90 participants in the reference study, 84 (93.3%) completed the two-year follow-up. Retention was 95.6% in the intervention group and 91.1% in the control group. Overall, six participants (6.7%) were lost to follow-up. The 6.7% loss to follow-up reported in this follow-up study was calculated based on the original sample size, N = 90 participants in the reference study [31].

## 3. Results

### 3.1. Socio-Demographic Characteristics of the Participants

Out of 90 students followed up for two years after SBE, the proportion of females was higher in the control group, 33 (80.5), compared to the intervention group, 22 (51.2). However, in relation to sex, there was a significant difference between the intervention and control groups. At the time of follow-up, 64.3% of nurse-midwives were employed whereas 35.7% were unemployed (Table 1).

### 3.2. Nurses-Midwives with Correct Knowledge Scores on INC to a Healthy Breathing Neonate at Post-Test and Follow-Up, Between Intervention and Control Groups (N = 84)

In the intervention group, knowledge of the definition of initial neonatal care declined significantly from 52.4% at post-intervention to 14.3% (*p* < 0.001) at follow-up. Significant declines were also observed in knowledge that wiping liquor/meconium prevents aspiration (97.7% to 72.1%, *p* = 0.003), that INC reduces aspiration risk (100.0% to 76.7%, *p* = 0.002) and that it prevents hypoglycaemia (97.7% to 83.7%, *p* = 0.031). Conversely, knowledge of mother-to-skin-to-skin contact increased significantly from 54.8% to 83.3% (*p* = 0.008). In the control group, significant improvements were observed in knowledge of drying to keep the neonate warm (37.5% to 90.0%, *p* < 0.001), skin-to-skin contact (10.0% to 80.0%, *p* < 0.001) and appropriate timing for initial neonatal care (29.3% to 63.4%, *p* = 0.004). However, knowledge on assessment of neonatal breathing declined significantly from 42.5% to 7.5% (*p* = 0.003), and knowledge that wiping liquor/meconium prevents aspiration declined from 85.4% to 29.3% (*p* < 0.001). Knowledge of hypothermia prevention remained at 100% in both groups (Table 2).

### 3.3. Nurse-Midwives with Correct INC Knowledge on Neonates with Difficulty Breathing at Post-Test and Follow-Up Between Intervention and Control Groups

Table 3 compares the post-intervention and follow-up findings. Neonatal resuscitation knowledge showed mixed changes at follow-up. Knowledge of the definition of neonatal resuscitation declined significantly in both the intervention group, from 93.0% to 30.2% (*p* < 0.001) and the control group, from 80.5% to 19.5% (*p* < 0.001). Recognition of difficult breathing as a serious neonatal condition also declined significantly in both groups, from 79.1% to 44.2% (*p* = 0.001) in the intervention group and from 92.7% to 29.3% (*p* < 0.001) in the control group. Conversely, knowledge that hypothermia is a serious neonatal condition increased significantly in the intervention group from 30.2% to 67.4% (*p* < 0.001) but did not change significantly in the control group. Knowledge of wiping the mouth and nose before stimulation declined significantly in the control group from 73.2% to 43.9% (*p* = 0.023). Knowledge of timely ventilation and correct mask placement remained high with no significant changes in either group.

### 3.4. INC Knowledge Gain and Retention

**Figure 2:**
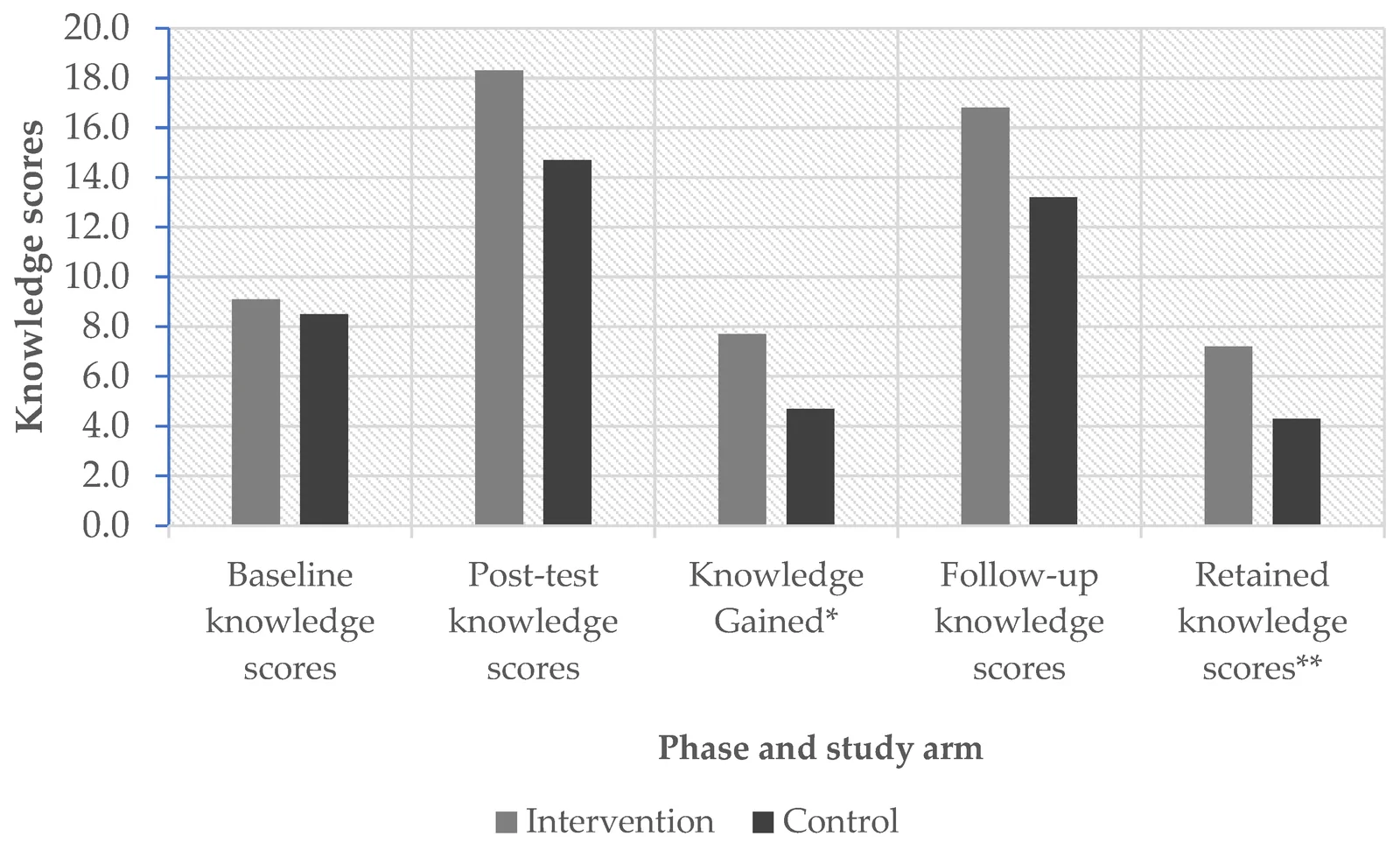
Changes in mean INC knowledge scores over time (baseline, post-test, and follow-up) among intervention and control groups. Note: * Knowledge gained was calculated by subtracting baseline knowledge scores from post-test knowledge scores, while ** retained knowledge score was calculated by subtracting baseline scores from follow-up knowledge scores. The maximum possible knowledge score was 22.

Knowledge gain reflects change in knowledge score from baseline to immediately post-intervention assessment. Knowledge decay refers to the reduction in knowledge score from the immediate post-intervention assessment to the two-year follow-up, whereas knowledge retention refers to the knowledge maintained at the two-year follow-up compared to the knowledge achieved immediately after the intervention. The overall trend of INC knowledge scores across baseline, post-test and two-year follow-up between intervention and control groups is shown in Figure 2. At baseline, the mean knowledge score was slightly higher in the intervention group (9.1) than in the control group (8.5). Immediately after the SBE, knowledge increased markedly in the intervention group (18.3) compared with the control group (14.7). Knowledge gain was therefore higher in the intervention group (9.2 points) than in the control group (6.2 points), as reported in the reference study [31]. At follow-up, knowledge scores declined in both groups but remained above baseline levels, with higher scores in the intervention group (16.3) than in the control group (12.7). Retained knowledge was also higher in the intervention group (7.2 points) compared with the control group (4.2 points). Overall, while both groups improved over time, the intervention group showed greater knowledge acquisition, higher gains, and better retention.

### 3.5. The Effect Size of SBE on INC Knowledge Retention (Difference-in-Difference (DID))

The results in Table 4 indicate no statistically significant long-term effect of SBE on INC knowledge retention. The unadjusted difference-in-differences estimate was 0.02 (95% CI: −1.68–1.67), *p* = 0.983, while the adjusted estimate was 0.06 (95% CI: −1.47–1.60), *p* = 0.935, after adjusting for gender, meaning that the mean reduction in knowledge scores was slightly greater in the control group, whereas in the intervention group, it was 0.06 points.

## 4. Discussion

This study evaluated the long-term effect of simulation-based education (SBE) on knowledge retention in initial neonatal care (INC) among the nurse-midwives in Kilimanjaro, Tanzania. By extending follow-up to two years, the study provides important insights into the durability of learning outcomes beyond the short-term gains commonly reported in the literature [23,41]. The study employed the same measurement points and outcome measures as in the reference study [31]. The same knowledge questionnaire used at the pre-test was used at the post-test and at follow-up to evaluate knowledge retention and estimate difference-in-differences (DiD).

This study demonstrated that at the two-year retention assessment, knowledge declined in both groups. Despite the decline, the intervention group retained higher knowledge scores than the control group and remained above baseline levels. The observed retention advantage (7.2 points vs. 4.2 points) indicates that although simulation fosters durable learning, it may not completely prevent forgetting [42]. The decline in knowledge is widely documented in many nursing education and healthcare worker studies, which report a decline in knowledge in helping babies breathe between 6 months and 1 year after training [29,30,43]. The current findings indicate that a single SBE, as conducted in our reference study [31], might be insufficient for sustaining knowledge over a long period of time after transitioning to nurse-midwives. Our study emphasizes periodic refresher training and frequent skills practice to sustain clinical competence and quality care delivery [41].

Importantly, while the intervention group performed better descriptively, the difference-in-differences results revealed no statistically significant long-term effect of SBE on knowledge retention over the two-year period. Evidence from educational theory demonstrates that long-term knowledge retention is strengthened by active learning, spaced repetition, and ongoing reinforcement rather than one-time exposure [44,45]. According to the forgetting curve by [46,47], newly acquired information declines rapidly when no active review is conducted to retain it. Therefore, incorporating periodic simulation sessions throughout nursing and midwifery education and early professional practice could strengthen memory consolidation and improve long-term competence. Spaced repetition counteracts the effect of forgetting and improves long-term retention of information by gradually expanding the repeated exposure sessions at increasing intervals to reinforce memory, making information harder to forget [45]. Therefore, the absence of statistical significance in the difference-in-difference analysis should be interpreted cautiously, as it may reflect insufficient reinforcement rather than the ineffectiveness of SBE.

The findings of this study showed that both groups declined in knowledge in many critical areas: assessment of neonatal breathing at birth, recognizing difficulty breathing as a serious situation, and initiation of ventilation within the first minute of birth. The decline in knowledge in these items is of particular concern, as it may compromise timely clinical decision-making for neonatal management. Delays in initiating ventilation increase the risk of prolonged asphyxia and long-term neurodevelopmental disabilities or neonatal mortality [48]. The probable reason for similar decline in knowledge in both groups may be due to a single SBE conducted in the reference study [31], which may not have been sufficient for the intervention to demonstrate a greater effect on knowledge retention than the lecture-based methods provided to the control group. WHO recommends assessment and immediate bag-mask ventilation for neonates with difficulty breathing to support and sustain breathing [15]. Our findings align with previous studies that report how both cognitive and psychomotor competencies can deteriorate after initial training when knowledge and skills are not regularly learned and practiced [49]. These findings highlight the importance of integrating periodic training and continuous professional development into both nursing and midwifery education and hospital protocols. 

Differences in employment status after graduation, opportunities for clinical exposure, particularly in maternity and labor wards, and differences between study settings represent potential contextual factors that may have influenced the observed findings because their contribution could not be collected in the current study. The questionnaire was retained from the previous study to maintain comparability of the key variables and knowledge outcomes between the two studies. However, repeated administration of the same 22-item knowledge questions at baseline, post-intervention and two-year follow-up may have introduced knowledge testing/practice effects, whereby participants became familiar with the questions or correct responses over time, potentially influencing scores independently of the intervention. During the first year of follow-up, students had exposure to INC in labor wards. By the second year of follow-up, all students graduated with more than sixty percent employed as nurse-midwives and the remainder unemployed or engaged in non-professional activities. Time to employment and differences in hands-on exposure among unemployed graduates may have contributed to the decline in knowledge. These findings highlight the importance of supporting the transition of graduates into the workforce with deployment in clinical practice environments where competencies acquired during training can be maintained and further developed for quality care delivery [41]. Furthermore, although the rural school had comparable characteristics to the intervention group, including the curriculum, enrolment capacity, and human resources, differences in school settings may also have influenced the findings. In Tanzania, diploma-level nursing and midwifery education follows similar standards across institutions, regardless of whether they are located in urban or rural settings. Nevertheless, contextual differences between urban and rural schools cannot be completely excluded as potential sources of variation in the observed findings.

Interestingly, INC knowledge related to thermal care, such as skin-to-skin contact and reducing risks of hypothermia, improved and remained high at follow-up in both groups. These findings could reflect frequent reinforcement of INC (essential newborn care and neonatal resuscitation) through global and national guidelines, diploma curriculum integration, and local protocols, providing frequent exposure and routine practice [10,36]. The improvements observed in the control group may reflect the effect of previous assessments, clinical exposure, subsequent learning, or curricular and institutional differences, highlighting the complementary role of clinical immersion alongside formal education [50]. These findings suggest that consistent exposure and repetition through both formal training and clinical experience are vital for sustaining knowledge.

Implication: Integrating simulation-based education into nursing curriculum and professional training may enhance knowledge retention.

### Strengths and Limitations of the Study

The strength of this study is its two-year follow-up period, offering rare evidence on long-term knowledge retention after SBE in low-resource settings. Few studies have examined educational outcomes over such a long period of time. Thus, these findings provide useful information for curriculum development and continuing professional education. Several limitations should be acknowledged: First, participants were recruited from two different nursing schools; the intervention was in an urban school and the comparison group in a rural school.

Differences in learning environments and institutional resources may have influenced educational outcomes independently of the intervention. Second, participants had different levels of clinical exposure after graduation. Graduate nurse-midwives employed might have been placed in maternal and labor wards, including labor wards where they may have reinforced their knowledge through routine INC practice compared to unemployed graduates. Nevertheless, differences in employment status, clinical placement, and duration of practice after employment could not be statistically assessed because data were not collected for these variables to ensure comparison with the reference study. Limited information on those variables limits understanding of whether the retained knowledge was independently contributed to the intervention. Third, the repeated administration of the same 22-item knowledge questionnaire at baseline, post-intervention and two-year follow-up may have introduced testing or practice effects, whereby participants became familiar with the questions or correct responses over time, potentially influencing scores independently of the intervention. Finally, this study evaluated knowledge retention but did not assess initial neonatal care skills practices and neonatal outcomes. Future research is required to assess whether retained knowledge translates into clinical skills to improve neonatal outcomes.

## 5. Conclusions

The intervention group had higher observed knowledge scores at follow-up, but the study did not demonstrate a statistically significant differential long-term effect of the single SBE intervention. These findings suggest that a single simulation experience is insufficient for sustained knowledge over time.

## Figures and Tables

**Table 1:** Demographic characteristics of students followed up two years after the SBE intervention (N = 84).

Characteristic	Control [n = 41 (%)]	Intervention [n = 43 (%)]	*p*-Value	Total [N = 84 (%)]
Gender				
Male	8 (19.5)	21 (48.8)	0.005	29 (34.5)
Female	33 (80.5)	22 (51.2)		55 (65.5)
Secondary education level				
Form 4	31 (75.6)	31 (72.1)	0.714	62 (73.8)
Form 6	10 (24.4)	12 (27.9)		22 (26.2)
Age in years, mean SD	24.17 ± 3.35	23.93 ± 1.74	0.679	24.05 ± 2.64
Age group				
<21 years	4 (9.8)	3 (7.0)	0.645	7 (8.3)
22 to 36 years	37 (90.2)	40 (93.0)		77 (91.7)
Marital status				
Married	4 (9.8)	6 (14.0)	0.553	10 (11.9)
Single	37 (90.2)	37 (86.0)		74 (88.1)
Currently employed as a nurse-midwife				
No	15 (36.6)	15 (34.9)	0.871	30 (35.7)
Yes	26 (63.4)	28 (65.1)		54 (64.3)

**Table 2:** Longitudinal comparison of correct initial neonatal care knowledge among nurses-midwives at post-test and follow-up by study groups using McNemar’s test.

	Intervention				Control			
Variable	Matched (n)	Post- Test, n (%)	Follow-Up, n (%)	*p*-Value	Matched (n)	Post-Test, n (%)	Follow-Up, n (%)	*p*- Value
Initial neonatal care	43	42 (97.7)	43 (100.0)	1.000	41	40 (97.6)	41 (100.0)	1.000
Definition of initial neonatal care	42	22 (52.4)	6 (14.3)	<0.001	39	10 (25.6)	4 (10.3)	0.146
Assessment of breathing	42	27 (64.3)	19 (45.2)	0.134	40	17 (42.5)	3 (7.5)	0.003
Drying to keep warm	42	35 (83.3)	38 (90.5)	0.549	40	15 (37.5)	36 (90.0)	<0.001
Mother-skin-to-skin contact	42	23 (54.8)	35 (83.3)	0.008	40	4 (10.0)	32 (80.0)	<0.001
Early initiation of breastfeeding	42	28 (66.7)	31 (73.8)	0.607	40	7 (17.5)	17 (42.5)	0.041
Delayed cord cut	42	13 (31.0)	10 (23.8)	0.629	40	1 (2.5)	3 (7.5)	0.625
Time of initial neonatal care is most important for a healthy breathing neonate	43	40 (93.0)	37 (86.0)	0.453	41	12 (29.3)	26 (63.4)	0.004
Wiping liquor and meconium-stained liquor prevents neonatal aspiration	43	42 (97.7)	31 (72.1)	0.003	41	35 (85.4)	12 (29.3)	<0.001
Dry, cover, skin-to-skin contact and early initiation of breastfeeding	43	43 (100.0)	43 (100.0)	NA	41	41 (100.0)	40 (97.6)	1.000
Time for APGAR Score	43	43 (100.0)	38 (88.4)	0.062	41	30 (73.2)	20 (48.8)	0.052
Initial neonatal care prevents neonatal hypothermia	43	43 (100.0)	43 (100.0)	NA	41	41 (100.0)	41 (100.0)	NA
Initial neonatal care reduces risks of aspiration	43	43 (100.0)	33 (76.7)	0.002	41	41 (100.0)	16 (39.0)	<0.001
Initial neonatal care initiates neonatal resuscitation	43	40 (93.0)	34 (79.1)	0.070	41	36 (87.8)	34 (82.9)	0.754
Initial neonatal care prevents neonatal hypoglycemia	43	42 (97.7)	36 (83.7)	0.031	41	37 (90.2)	25 (61.0)	0.012

Note: The follow-up analysis included 43 matched participants in the intervention group and 41 in the control group. Item-specific matched denominators varied because of missing responses at one or both assessment time points. For each item, analyses were restricted to participants with valid paired responses at both post-test and follow-up. Percentages were calculated using the corresponding item-specific matched denominator. Changes in paired binary responses were assessed using McNemar’s exact test. NA indicates that no discordant pairs were observed and therefore a McNemar test statistic and *p*-value could not be estimated.

**Table 3:** Longitudinal comparison of correct neonatal resuscitation knowledge among nurses-midwives at post-intervention and follow-up by study group (N = 84).

	Intervention				Control			
Variable	Matched (n)	Post- Test, n (%)	Follow-Up, n (%)	*p*-Value	Matched (n)	Post-Test, n (%)	Follow-Up, n (%)	*p*-Value
Neonatal resuscitation	43	43 (100.0)	42 (97.7)	1.000	41	40 (97.6)	41 (100.0)	1.000
Definition of neonatal resuscitation	43	40 (93.0)	13 (30.2)	<0.001	41	33 (80.5)	8 (19.5)	<0.001
Difficult breathing is a serious situation in immediately born neonates	43	34 (79.1)	19 (44.2)	0.001	41	38 (92.7)	12 (29.3)	<0.001
Hypothermia is a serious situation in neonates	43	13 (30.2)	29 (67.4)	<0.001	41	16 (39.0)	21 (51.2)	0.383
If the neonate is not breathing at birth due to secretions, the mouth and nose must be wiped before stimulating	43	40 (93.0)	38 (88.4)	0.727	41	30 (73.2)	18 (43.9)	0.023
Ventilation should be initiated within one minute of birth for a neonate with difficulty breathing	43	43 (100.0)	39 (90.7)	0.125	41	38 (92.7)	32 (78.0)	0.07
During ventilation, the face mask must cover the nose, mouth and chin	43	43 (100.0)	41 (95.3)	0.500	41	39 (95.1)	36 (87.8)	0.453
Neonatal resuscitation	43	43 (100.0)	42 (97.7)	1.000	41	40 (97.6)	41 (100.0)	1.000

**Table 4:** Effect size of SBE on INC knowledge retention using Generalized Linear Mixed Model GLMM (N = 84).

Domain	uDiD, 95% CI	*p*-Value	aDiD, 95% CI	*p*-Value
Knowledge	0.02 (−1.68–1.67)	0.983	0.06 (−1.47–1.60)	0.935

Unadjusted difference-in-differences (uDiD) and adjusted difference-in-differences (aDiD). Gender is the only variable included in the adjusted model.

## Data Availability

Data analyzed for this study are available from the corresponding author upon reasonable request due to privacy or ethical restrictions.

## Supplementary Materials

The following supporting information can be downloaded at: https://www.mdpi.com/article/10.3390/nursrep16100365/s1, File S1: TREND reporting checklist; File S2: STROBE reporting check list.

## Public Involvement Statement

No public involvement in any part of this research.

## Guidelines and Standards Statement

This manuscript was drafted in accordance with the Transparent Reporting of Evaluations with Non-randomized Designs (TREND) for quasi-experimental studies [51] and STROBE (Strengthening the Reporting of Observational Studies in Epidemiology) for cohort studies: https://www.equator-network.org/wp-content/uploads/2015/10/STROBE_checklist_v4_cohort.pdf (accessed on 1 August 2026) (Supplementary Files S1 and S2).

## Abbreviations

The following abbreviations are used in this manuscript:

DiD	Difference-in-differences
HCPs	Healthcare providers
INC	Initial neonatal care
NaTHREC	Tanzania National Health Research Ethics Committee
NLN	Nursing League of Nursing
SBE	Simulation-based education
WHO	World Health Organisation
