# Supplementary material for: Long-Term Effect of Simulation-Based Education on Knowledge Retention in Initial Neonatal Care Among Nurse-Midwives in Kilimanjaro, Tanzania: A Quasi-Experimental Study
Source: Nurs Rep. 2026 Oct 15;16(10):365. doi: 10.3390/nursrep16100365 (PMC13708147; doi:10.3390/nursrep16100365)
Supplement: Supplementary file 1 [file nursrep-16-00365-s001.zip › Checklists for study assessment.zip!/STROBE_checklist for cohort studies.pdf]

STROBE Statement—Checklist of items that should be included in reports of ***cohort studies***

	Item No	Recommendation
**Title and abstract**	1	*(a)* Title: Long-term effect of simulation-based education on knowledge retention in initial neonatal care among nurse-midwives in Kilimanjaro, Tanzania: A quasi-experimental study<br>*(b)* This study assessed two-year knowledge retention following simulation-based education (SBE) on initial neonatal care among nurse-midwives in Tanzania. A quasi-experimental reference study was followed up after two years, comparing participants who received lectures plus SBE with those who received lectures alone. Data were collected using the same structured questionnaire used in the reference study and analysed using descriptive and inferential statistical methods, including Generalised Linear Mixed Models. The study showed that at two years after the intervention, knowledge scores declined in both groups, although the intervention group had higher observed knowledge scores and retention than the control group. However, the adjusted analysis found no statistically significant long-term effect of simulation-based education on knowledge retention.
**Introduction**		
Background/rationale	2	Simulation-based education is reported to improve nursing and midwifery students' clinical competencies, however, evidence regarding long-term retention of knowledge remains scarce, particularly in low-resource settings. This study evaluated two-years effect of simulation-based education on knowledge retention in initial neonatal care among nurse-midwives in Tanzania.
Objectives	3	Specific objective<br>The aim of this study was to evaluate the long-term effect of simulation-based education on knowledge retention in initial neonatal care among nurse-midwives in Tanzania.<br>Hypothesis: The study hypothesized that nurse-midwives receiving simulation-based education have higher long-term knowledge retention of initial neonatal care than those receiving lecture teaching methods only.
**Methods**		
Study design	4	The key elements of study design presented early in the paper are:<br>I. Intervention - Simulation-based education (SBE)<br>II. Study population: Nurse-midwives<br>III. Outcome: knowledge retention<br>IV. Topic / area: Initial neonatal care<br>V. Period/ time: long-term / two years
Setting	5	Setting: Participants originated from two nursing and midwifery schools; the urban and a rural school. After graduation, the participants were dispersed into different geographical settings: others employed, and others unemployed busy with individual activities.<br>Recruitment: recruitment was conducted in June 2025<br><br>Exposure: Both the interventional group and the control groups had 2-hour session of initial neonatal care (INC) lecture teaching in the classrooms. The intervention group received additional training in SBE.<br><br>Follow-up: The was a 2-year follow-up which started after post-intervention knowledge assessment in June 2023 to June 2025<br><br>Data collection: The same structured questionnaire used in the reference quasi-experimental study was employed. Participants were contacted using mobile phone, their responses were filled online in the electronic structured questionnaire.
Participants	6	(*a*) Give the eligibility criteria, and the sources and methods of selection of participants. Describe methods of follow-up

		Participants were 90 second-year students who participated in the reference study; 45 in the intervention and 45 students in the control group and followed up for two years after SBE. The study groups with INC lecture information but with additional SBE in the interventional group.<br>*(c)* **The study was not matched**<br>*(d)* NA
Variables	7	The primary outcome variable was knowledge retention in INC (comparing knowledge scores between the immediate post-intervention assessment and the two-year follow-up.<br>A secondary outcome variable was the intervention effect estimated by difference-in-differences (DiD) approach. Potential confounders were comparing knowledge retained from urban versus rural settings. Despite that both rural and urban schools' enrolment capacity, and curriculum are comparable, their learning characteristics may confound the observed findings
Data sources/ measurement	8*	The assessment methods were comparable between the study groups. The same questionnaire used in the reference study was retained in the current study to ensure comparability of the key variables and knowledge outcomes. The same knowledge domains, questions, and scoring approach were therefore used to assess the outcome, allowing the observed differences in knowledge to be compared across the study groups. No changes were made to the core assessment items that would have affected the measurement of the primary knowledge outcome.
Bias	9	Using the same questionnaire to reduce potential respondent bias.
Study size	10	Sample size calculation was based on the pre-test - post- intervention reference study and did not specifically account for the two-year DiD analysis (Malya et al., 2025). However, the current study was designed as a cohort follow-up of all the 90 eligible students who participated in the reference study during the two-year follow-up period. Therefore, 84 nurse-midwives included in the current study was determined by the number of 90 eligible graduates available during follow-up period, rather than by a statistical sample-size calculation.
Quantitative variables	11	Explain how quantitative variables were handled in the analyses. If applicable, describe which groupings were chosen and why
Statistical methods	12	McNemar's exact test, paired t-test<br>(b) Generalised Linear Mixed Model<br>(c) Participants were traced by phone, and recruitment was conveniently to those who were reached and consented for study participation.<br>(*e*) NA
**Results**		
Participants		13*<br>• 90 second-year students: 45 from the intervention and 45 in the control group participated in the reference study.<br>• Those students were followed up for two years and graduated as nurse-midwives.<br>• At the time of recruitment, 84:43 in the intervention and 41 in the control group were recruited in the follow-up study<br>• A flow diagram showing the 90 participants in the reference study, 84 who completed the two-year follow-up, and loss to follow-up indicated in the main text.<br>• Among the 90 participants in the reference study, 84 who completed the two-year follow-up, and loss to follow-up indicated in the main text<br>• Generalized Linear Mixed Model (GLMM) adjusted for gender only<br>•

**Discussion**		
Key results	18	After two years of simulation-based education on initial neonatal care, knowledge declined in both groups but remained higher in the intervention group. The adjusted difference-in-difference analysis showed no significant long-term effect of simulation on knowledge retention.
Limitations	19	Differences in school settings, different levels of clinical exposure after graduation may have influenced educational outcomes independently of the intervention. Nevertheless, since differences in employment status, clinical placement and duration of practice after employment could not statistically be assessed because data were not collected for these variables to ensure comparison with the reference study.
Interpretation	20	The intervention may have contributed to the observed knowledge retention, but the study cannot completely separate its effect from post-graduation employment, clinical exposure, duration of practice, and differences in learning/institutional environments.
Generalisability	21	Based on the statements you provided, the generalisability of the findings may be limited because the study participants were exposed to different learning environments, employment circumstances, and opportunities for clinical practice after graduation. These factors may differ substantially across schools and could influence knowledge retention independently of the intervention.
**Other information**		
Funding	22	This study was funded by the Norwegian Agency for Development Cooperation (NORAD) through the NORHED II project, Implementation of Simulation-Based Education in Tanzania and Malawi [Grant No. 70722].<br><br>Malya, R. M., Urstad, K. H., Bø, B., Mahande, M. J., & Rogathi, J. (2025). Simulation-based education on initial neonatal care knowledge among nursing and midwifery students in Tanzania: Quasi experimental study. *International Journal of Africa Nursing Sciences*, *22*(March), 1–8. https://doi.org/10.1016/j.ijans.2025.100847

*Give information separately for exposed and unexposed groups.

Note: An Explanation and Elaboration article discusses each checklist item and gives methodological background and published examples of transparent reporting. The STROBE checklist is best used in conjunction with this article (freely available on the Web sites of PLoS Medicine at http://www.plosmedicine.org/, Annals of Internal Medicine at http://www.annals.org/, and Epidemiology at http://www.epidem.com/). Information on the STROBE Initiative is available at http://www.strobe-statement.org.
