# Supplementary material for: Long-Term Effect of Simulation-Based Education on Knowledge Retention in Initial Neonatal Care Among Nurse-Midwives in Kilimanjaro, Tanzania: A Quasi-Experimental Study
Source: Nurs Rep. 2026 Oct 15;16(10):365. doi: 10.3390/nursrep16100365 (PMC13708147; doi:10.3390/nursrep16100365)
Supplement: Supplementary file 1 [file nursrep-16-00365-s001.zip › Checklists for study assessment.zip!/TREND_Reporting guideline and Standard Report.pdf]

TREND: WRITING REPORTS FOR QUASI-EXPERIMENTS

by Simon Moss

Introduction

The TREND statement—an acronym that represents the Transparent Reporting of Evaluations with Non-randomized Designs—is a checklist writers should utilize when they report a quasi-experiment. To clarify, researchers often compare two or more conditions, such as two teaching methods. In many of these studies, the participants or units are not randomly assigned to these conditions. This design is called a quasi-experiment or non-randomized design. TREND is a set of principles that researchers should observe whenever they report quasi-experiments or non-randomized designs.

Illustration

Suppose a researcher wants to explore whether lecturers who wear casual attire enhances student engagement more than do lectures who wear formal attire. In this study, students are not randomly allocated to these lecturers. Instead, whether they observe lecturers in casual attire or formal attire depends on whether they attended Tuesday classes or Wednesday classes.

Purpose of this document

This document lists every TREND principle and then includes examples to illustrate each principle. These principles do not encompass every paragraph you should include. Instead, these principles stipulate the most important details you should include.

TREND checklist

Title and abstract		
Item	Description of the item—as specified by Des Jarlais et al (2004)	Example
1 Title and abstract	Information on how unit were allocated to interventions	• Title: Long-term effect of simulation-based education on knowledge retention in initial neonatal care among

		nurse-midwives in Kilimanjaro, Tanzania: A quasi-experimental study<br><br>• The title identifies the intervention (simulation-based education), the outcome (knowledge retention), the topic (initial neonatal care), and the study population (nurse-midwives) and time (long-term period – a two - year follow-up)<br>• The study population were nurse-midwives who were second-year diploma in nursing and midwifery students when they participated in a reference quasi experimental study between March to June 2023.<br>• In the reference study, 90 students were purposively recruited; 45 in the intervention group and 45 to the control group.<br>• The students were recruited purposively from two out of six schools offering diploma in nursing and midwifery in Kilimanjaro.<br>• During the two years of follow-up, students graduated as nurse-midwives and were dispersed in different geographical locations; others employed and others unemployed busy with individual activities.<br>• Out of 90 students who were followed up post-intervention from the reference study; 84: 43 in the intervention and 41 to the control groups were recruited in the follow-up study.<br>• Simulation was repeated and roles exchanged to maximize learning<br>• Both INC training and SBE intervention were facilitated by the researcher

	Structured abstract recommended	• The abstract presents the background, methods, results, and conclusion/implication of the study.
	Information on target population or study sample	• Out of 90 second-year diploma in nursing and midwifery students who participated in the reference- quasi-experimental study and graduated as nurse-midwives during a followed up, 84 nurse-midwives participated in the study.

Introduction		
Item	Description of the item	Example
2 Background	Scientific background and explanation of rationale	• Simulation-based education is reported to improve nursing and midwifery students' clinical competencies, however, evidence regarding long-term retention of knowledge remains scarce, particularly in low-resource settings. This study evaluated two-years effect of simulation-based education on knowledge retention in initial neonatal care among nurse-midwives in Tanzania.
	Theories used in designing behavioural interventions	• National League for Nursing (NLN) Jeffries Simulation Theory was used to design and implement and evaluating SBE

Method		
Item	Description of the item	Example
3 Participants	Eligibility criteria for participants, including criteria at different levels in recruitment/sampling plan (e.g., cities, clinics, subjects)	• All the second-year diploma in nursing and midwifery students who participated in the reference quasi-experimental study, followed up for two year, graduated as nurse-midwives access, and consented for study participation
	Method of recruitment (e.g., referral, self-selection), including the sampling method if a	• The researcher contacted the participants by mobile phone. Those who were successfully reached were informed about

	systematic sampling plan was implemented	the study procedures, provided written consents, and were conveniently recruited to in the study
	Recruitment setting	• Participants originated from two nursing and midwifery schools; the urban and a rural school.<br>• After graduation, all the participants were dispersed into different geographical settings: others employed, and others unemployed busy with individual activities
	Settings and locations where the data were collected	• Data was collected between 01 to 30 June 2025 electronically using the same structured questionnaire used in the reference study.<br>• The researcher and research assistant filled the digitally questionnaire to reduce risks of respondent bias.
4 Interventions	Details of the interventions intended for each study condition and how and when they were actually administered, specifically including	• The reference quasi-experimental study involved two groups; intervention with 45 students and a control group with 45 students.<br>• Simulation-based education intervention was conducted in June 2023 among intervention group with 45 students from urban school
	Content: what was presented?<br><br>Delivery method: how was the content presented?<br><br>Unit of delivery: how were the subjects grouped during delivery<br><br>Setting: where was the intervention delivered<br><br>Deliverer: who delivered the intervention?	• Before SBE, a two-hour auditorium lecture teaching was conducted on initial neonatal care (INC) among study groups.<br>• The intervention group received additional SBE in INC<br>• Two different INC scenarios were simulated; (1) INC for a normal, healthy-breathing neonate, and (2) INC for neonates with difficulty breathing at birth using a Baby NeoNatalie simulator.<br>• In a group of six to seven students, three participated in the experience serving as nurse-midwives, and two as observers for

		skills practice and communication within the team<br>• SBE was conducted in the simulation skills lab which is the lab owned by the university nearby the urban school of nursing where the interventional group students were drawn.<br>• Both INC lecture teaching and SBE were facilitated by the researcher
	Exposure quantity and duration: how many sessions or episodes or events were intended to be delivered? How long were they intended to last?<br><br>Time span to deliver the intervention to each unit?	• The first and second scenarios were simulated for 8 and 5 minutes respectively.<br>• Each student participated in two simulated scenarios.<br>• The debriefing session was conducted immediately after each simulated scenario, each lasting for 50 mins
	Activities to increase compliance or adherence (e.g., incentives)	• In the debriefing application phase, students were asked to summarize the key important messaged they had learned in the simulation and specify how they would do differently apply if they encountered a similar situation in the real future situations<br>• There is no additional SBE training conducted during follow-up period.
5 Objectives	Specific objectives and hypotheses	• The aim of this study was to evaluate the long-term effect of simulation-based education on knowledge retention in initial neonatal care among nurse-midwives in Tanzania.<br>• The hypothesis was that nurse-midwives receiving simulation-based education have higher long-term knowledge retention of initial neonatal care than those receiving lecture teaching methods only.
6 Outcomes	Clearly defined primary and secondary outcome measures	• The primary outcome variable was knowledge retention in INC (by comparing knowledge scores maintained between the post-intervention assessment and the two-year follow-up.

		• A secondary outcome variable was the intervention effect estimated by difference-in-differences (DiD) approach
	Methods used to collect data and any methods used to enhance the quality of measurements	• Data were collected in June 2025 using electronic structured questionnaire, the same structured questionnaire used at the pre-test and post-intervention in the reference study.<br>• Using a mobile phone the participants' responses were completed by the researcher and research assistant.
	Information on validated instruments such as psychometric and biometric properties	• The study data collection tool was developed with reference to established standards and guidelines, including the Tanzanian Nursing and Midwifery Curriculum, National Neonatal Care Guidelines and relevant World Health Organization recommendations. The tool was subsequently piloted, validated and standardized before its use in the study. Its psychometric properties were assessed, including internal consistency using Cronbach's alpha, while evidence of content, construct, criterion and convergent validity was considered as appropriate.
7. Sample size	How sample size was determined and, when applicable, explanation of any interim analyses and stopping rules.	• The sample size was not calculated in the current study, but using an intervention-study formula based on 5% significance, 80% power, anticipated proportions of 62.7% and 86.7%, and 10% attrition. The resulting sample was 45 participants per group, giving a total of 90 participants. No interim analyses or stopping rules were planned.
8 Assignment method	Unit of assignment (the unit being assigned to study condition, e.g., individual, group, community)	• Assignment of participants were at the group and school level. The group assignment was made; one group assigned in the intervention and the other in the control group
	Method used to assign units to study conditions, including details	• At the second- academic year of studying diploma in nursing and midwifery program

	of any restriction (e.g., blocking, stratification, minimization)	(this is according to the curriculum), students received 2-hour lecture sessions on initial neonatal care from their classrooms and before clinical rotation practice.<br>• Thus, the second-year students were purposively enrolled in the study (reference quasi-experimental study) because they had exposure to theoretical knowledge in INC before initiation of the reference study.<br>• Moreover, at this follow-up study, the graduated nurse-midwives were purposively recruited in the study but at their conveniently pace of being accessible.<br>• In this follow-up study, purposive recruitment was used, but participants were recruited when they were conveniently reachable
	Inclusion of aspects employed to help minimize potential bias induced due to non-randomization (e.g., matching)	• To minimize potential bias and the risk of contamination associated with non-randomization, participants were purposively recruited from two different schools. The intervention and control groups were recruited from separate schools to reduce the information contamination to the intervention between groups.<br>• Additionally, the control group had comparable characteristics to the intervention group, making it easier to have equal number of participants.
9. Blinding	Whether or not participants, those administering the interventions, and those assessing the outcomes were blinded to study condition assignment; if so, statement regarding how the blinding was accomplished and how it was assessed.	• To diminish demand characteristics and similar biases, the person administering the intervention—the lecturer—was not informed of the study hypotheses.<br>NA
10 Unit of analysis	Description of the smallest unit analyses to assess intervention	• The engagement of each individual nurse-midwife was included in the analyses

	effects (e.g., individual, group, or community)	
	If the unit of analysis differs from the unit of assignment, the analytical method used to account for this (e.g., adjusting the standard error estimates by the design effect or using multilevel analysis)	NA
11 Statistical analysis	Statistical methods used to compare study groups for primary methods outcome(s), including complex methods of correlated data	• A generalized linear mixed-effects model (GLMM) with a Gaussian distribution and identity link was used to analyze the approximately continuous knowledge score.<br>• A difference-in-differences approach compared changes from post-intervention to two-year follow-up between intervention and control groups. Models included study phase, study group and their interaction, with gender as the only adjustment covariate due to baseline imbalance. A random participant intercept accounted for repeated measurements. Effects were reported as β coefficients with 95% confidence intervals and statistical significance was assessed at the 5% level.
	Statistical methods used for additional analyses, such as a sub-group analyses and adjusted analysis	• Gender was retained as the adjustment covariate because it was the only characteristic showing evidence of imbalance between study groups. No subgroup analyses were specified or conducted.
	Methods for imputing missing data, if used	• All the 84 participants' data were included in the analyses, unlike for the missing data
	Statistical software or programs used	STATA version 17 (Stata Corp, College Station, TX, USA) was utilized to conduct these analyses.<br>•

Results

Item	Description of the item	Example
12 Participant flow [Often include a diagram]	Enrolment: the numbers of participants screened for eligibility, found to be eligible or not eligible, declined to be enrolled, and enrolled in the study<br><br>Assignment: the numbers of participants assigned to a study condition<br><br>Allocation and intervention exposure: the number of participants assigned to each study condition and the number of participants who received each intervention	• Out of 90 students who participated in the reference study, 84 graduate nurse-midwives; 43 from intervention and 41 in the control groups were enrolled in this follow-up study<br>• The participants flow diagram showing transition of students to nurse-midwives, number of enrolments is available in the main text.
	Follow-up: the number of participants who completed the follow- up or did not complete the follow-up (i.e., lost to follow-up), by study condition	• Out of 90 students who participated in the reference study and graduated as nurse-midwives, 84 nurse-midwives completed the follow-up study
	Analysis: the number of participants included in or excluded from the main analysis, by study condition	• NA
	Description of protocol deviations from study as planned, along with reasons	• NA
13 Recruitment	**Dates defining the periods of recruitment and follow-up**	• The first recruitment of these participants was in March 2023 when the pre-test INC knowledge was assessed, followed by post-intervention knowledge assessment in June 2023. The last recruitment was in June 2025 for a follow -up study.
14 Baseline data	Baseline demographic and clinical characteristics of participants in each study condition.	• Table 1 presents the mean age, the percentage of males and females, marital status, educational level, and employment status of the participants
	Baseline characteristics for each study condition relevant to	• NA

	specific disease prevention research	
	Baseline comparisons of those lost to follow-up and those retained, overall and by study condition	• NA
	Comparison between study population at baseline and target population of interest	• NA
15 Baseline equivalence	Data on study group equivalence at baseline and statistical methods used to control for baseline differences	• Table 1 indicate that the proportion of fe-males was higher in the control group compared to the intervention group<br>• However, in relation to sex, there was a significant difference between the intervention and control groups.<br>• At the time of follow-up, more nurse-midwives were employed compared unemployed<br>•
16 Numbers analysed	Number of participants (denominator) included in each analysis for each study condition, particularly when the denominators change for different outcomes; statement of the results in absolute numbers when feasible	• The analysis included 84 participants, comprising 43 in the intervention group and 41 in the control group. Denominators varied across outcomes and assessment time points because of missing observations. Results were reported using absolute numbers where feasible, with the corresponding denominators clearly indicated for each analysis.
	Indication of whether the analysis strategy was "intention to treat" or, if not, description of how non-compliers were treated in the analyses	• The analysis followed a per-protocol approach, whereby participants were analysed according to the predefined study procedures and intervention requirements. All participants who completed the intervention adhered to the study protocol and completed the required follow-up assessments. However, participants who could not be reached by telephone for follow-up were excluded from the per-protocol analysis. These participants accounted for less than 10% of the sample, which was within the 10% non-response rate anticipated during the sample size

		calculation. No participants were excluded because of protocol deviations or non-compliance.
17 Outcomes and estimation	For each primary and secondary outcome, a summary of results for each estimation study condition, and the estimated effect size and a confidence interval to indicate the precision	• Figure 1 showed that knowledge increased in the intervention group immediately post-intervention compared with the control group. while at follow up, knowledge scores declined in both groups but remained above higher in the intervention than in the control group.<br>• Table 4 showed non-significant difference-in-difference (DiD)- aDiD = 0.06; 95% CI: -1.47 to 1.60; P = 0.935)<br>• Hence, gender was controlled in the mixed effects regression model.<br>•
	Inclusion of null and negative findings	• NA
	Inclusion of results from testing pre-specified causal pathways through which the intervention was intended to operate, if any	• NA
18 Ancillary analyses	Summary of other analyses performed, including subgroup or restricted analyses, indicating which are pre-specified or exploratory	• NA
19 Adverse events	Summary of all important adverse events or unintended effects in each study condition (including summary measures, effect size estimates, and confidence intervals)	• No important adverse events or unintended effects were reported in either the intervention or control group during the study period. Consequently, no effect size estimates or confidence intervals were calculated for adverse events.

Discussion		
Item	Description of the item	Example

20 Interpretation	Interpretation of the results, taking into account study hypotheses, sources of potential bias, imprecision of measures, multiplicative analyses, and other limitations or weaknesses of the study	• The findings were interpreted in relation to the study hypothesis and the observed changes in outcomes between the intervention and control groups. The results should be considered in light of potential sources of bias, including loss to follow-up among participants who could not be reached by telephone and the possibility of residual confounding. The relatively small sample size may have reduced statistical precision and contributed to wider confidence intervals and convergence difficulties in adjusted models. The use of a per-protocol analysis may also introduce selection bias if participants who remained in the analysis differed systematically from those lost to follow-up. In addition, the study may have had limited power to detect smaller intervention effects or differences across subgroups. These limitations were considered when interpreting the magnitude, direction and statistical significance of the observed intervention effects and reported as limitation of the study.
	Discussion of results taking into account the mechanism by which the intervention was intended to work (causal pathways) or alternative mechanisms or explanations	Importantly, while the intervention group performed better descriptively, the difference-in-difference results revealed no statistically significant long-term effect of SBE on knowledge retention over the two-year period. incorporating periodic simulation sessions throughout nursing and education and early professional practice could strengthen memory consolidation and im-prove long-term competence
	Discussion of the success of and barriers to implementing the intervention, fidelity of implementation	• NA
	Discussion of research, programmatic, or policy implications	• NA

21 Generalizability	Generalizability (external validity) of the trial findings, taking into account the study population, the characteristics of the intervention, length of follow-up, incentives, compliance rates, specific sites/settings involved in the study, and other contextual issues	• The strength of this study is the long-term study conducted in low-resource settings.<br>• These findings provide insights on the benefits of implementing SBE, highlighting that SBE with no reinforcement potentially ends with knowledge loss<br>• Since SBE is a new technology in these settings, the findings of this study are valuable for implementation of SBE in these contexts' nursing schools to enhance students' knowledge, prepare them for professional life and improve quality of neonatal care
22 Overall evidence	General interpretation of the results in the context of current evidence and current theory	• These results demonstrate that had higher knowledge scores at follow-up among intervention group, but the study did not demonstrate a statistically significant differential long-term effect of the single SBE intervention.<br>• The findings suggests that a single simulation experience is insufficient for sustained knowledge overtime.<br>•

References

Des Jarlais, D. C., Lyles, C., Crepaz, N., & the Trend Group (2004). Improving the reporting quality of nonrandomized evaluations of behavioral and public health interventions: The TREND statement. American Journal of Public Health, 94, 361-366. For more information, visit: http://www.cdc.gov/trendstatement/
