# Supplementary material for: Long-Term Effect of Simulation-Based Education on Knowledge Retention in Initial Neonatal Care Among Nurse-Midwives in Kilimanjaro, Tanzania: A Quasi-Experimental Study
Source: Nurs Rep. 2026 Oct 15;16(10):365. doi: 10.3390/nursrep16100365 (PMC13708147; doi:10.3390/nursrep16100365)
Supplement: Supplementary file 1 [file nursrep-16-00365-s001.zip › INC Questionnaire.zip!/Experts comments on a questionnaire.pdf]

Comments - Questionnaire review by experts:

Question 4. "Which semester of learning are you"

Question 5. "Year of study"

Reviewer 1: Delete as it has no contribution to knowledge assessment.

Reviewer 3: Indicating year of study could capture the required for what is the study intending to assess rather than semester of learning. However, it is not clear whether it will be clear and under stable by saying 'year of study'

Reviewer 2: Remove both 4&5 as it's clear that the potential study participants / study population is will be students with theoretical knowledge in initial neonatal care

Conclusion: Question 4 and 5 may be deleted unless the research have any other scientific reasons.

Question 8: Are you interested in studying nursing and midwifery course?

Reviewer 1: Delete this question. I am curious: how is it possible for a second-year student not to be interested in the course?

Reviewer 2 &3: The question has no relevance with the initial neonatal care knowledge research

Question 15: If neonate is not breathing after birth due to secretion, mouth and nose should be wiped, suctioned, before stimulating

Reviewer 1: what kind of suction? penguin? May be penguin

Reviewer 2: Suction tube for neonatal resuscitation does not apply in the current practice

Reviewer 3: either of the penguin sucker or suction tube is recommended for basic neonatal resuscitation
