# Supplementary material for: Long-Term Effect of Simulation-Based Education on Knowledge Retention in Initial Neonatal Care Among Nurse-Midwives in Kilimanjaro, Tanzania: A Quasi-Experimental Study
Source: Nurs Rep. 2026 Oct 15;16(10):365. doi: 10.3390/nursrep16100365 (PMC13708147; doi:10.3390/nursrep16100365)
Supplement: Supplementary file 1 [file nursrep-16-00365-s001.zip › INC Questionnaire.zip!/Pilot tested INC questionnaire.pdf]

QUESTIONNAIRE

INITIAL NEONATAL CARE KNOWLEDGE ASSESSMENT

Participant ID:

Date:

Instructions: For questions requiring a Yes/No response, select either "Yes" or "No." For other questions, write your answer in the space provided.

Section A: Socio- demographic Characteristics

1. Gender of respondent
   ☐ Male
   ☐ Female
2. What is your age?
   Response: ___________________________________
3. Secondary educational level
   ☐ Form 4
   ☐ Form 6
4. Do you have previous exposure in simulation-based education training?
   ☐ No
   ☐ Yes
5. If YES; Where did you get the simulation training?
   ☐ From the school of nursing sessions
   ☐ From the research study
   ☐ Other source
6. Are you transferred from other nursing training school
   ☐ No
   ☐ Yes
7. Marital status
   ☐ Married
   ☐ Cohabiting

☐ Single
☐ Divorced

Section B: Knowledge on initial Neonatal Care for a healthy breathing neonate:

8. Have you ever heard of Initial Neonatal Care? *

   ☐ No
   ☐ Yes

9. Define Initial Neonatal Care

10. List 5 elements of initial neonatal care for a healthy breathing neonate:

Timing and priority of initial neonatal care provided to a healthy neonate after birth:

11. At what time after birth is initial neonatal care considered most important for the neonate?

   ☐ Within one hour after birth
   ☐ Within 24 hours

12. Wiping liquor and meconium-stained liquor from mouth and nose while the head appears at the vulva prevent neonatal aspiration *

   ☐ No
   ☐ Yes

13. If the neonate is breathing well after full delivery, dry, cover with dry cloth, apply skin-to-skin contact to prevent hypothermia and early initiation of breastfeeding

   ☐ No
   ☐ Yes

14. What time is APGAR Score first performed

   ☐ 1 and 10 minutes
   ☐ 1 and 5 minutes

Benefits of initial neonatal care (INC) to a healthy breathing neonate.

15. Initial neonatal care practices prevent neonatal hypothermia

   ☐ No

☐ Yes

16. Initial neonatal care practices reduce risks of aspiration

☐ No

☐ Yes

17. Initial neonatal care evaluation Initiates neonatal resuscitation

☐ No

☐ Yes

18. Initial neonatal care practices prevent neonatal hypoglycemia

☐ No

☐ Yes

Knowledge on neonatal resuscitation **(neonates with difficulty breathing at birth)**

19. Have you heard about neonatal resuscitation?

☐ No

☐ Yes

20.What do you understand about neonatal resuscitation?

____

____

21. If neonate is not breathing after birth due to secretion, mouth and nose should be wiped before stimulating

☐ No

☐ Yes

22.Ventilation should be initiated within one minute of birth to a neonate with difficulty breathing

☐ No

☐ Yes

23. During ventilation, face mask should must cover nose, mouth and chin

☐ No

☐ Yes
