# Supplementary material for: Long-Term Effect of Simulation-Based Education on Knowledge Retention in Initial Neonatal Care Among Nurse-Midwives in Kilimanjaro, Tanzania: A Quasi-Experimental Study
Source: Nurs Rep. 2026 Oct 15;16(10):365. doi: 10.3390/nursrep16100365 (PMC13708147; doi:10.3390/nursrep16100365)
Supplement: Supplementary file 1 [file nursrep-16-00365-s001.zip › INC Questionnaire.zip!/Questionnaire - Reviewed by experts.pdf]

QUESTIONNAIRE FOR INITIAL NEONATAL CARE KNOWLEDGE ASSESSMENT

**Participant identification number**	____
**Date of interview**	____ / ____ / __________

Instructions: **For questions requiring a Yes/No response, select either "Yes" or "No." For other questions, write your answer in the space provided.**

Section A: Socio- demographic Characteristics

1. **Gender of respondent**
   ☐ Male
   ☐ Female
2. **What is your age?**
   Response: ___________________________________
3. **Secondary educational level**
   ☐ Form 4
   ☐ Form 6
4. Which semester of learning are you?
   ____
5. **Year of study in nursing**
   ____
6. **Do you have previous exposure in simulation-based education training?**
   ☐ No
   ☐ Yes
7. **If YES; Where did you get the simulation training?**
   ☐ From the school of nursing sessions
   ☐ From the research study
   ☐ Other source
8. **Are you interested in studying nursing and midwifery course?**
   ____
9. **Are you transferred from other nursing training school**
   ☐ No
   ☐ Yes

10. **Marital status**

☐ Married

☐ Cohabiting

☐ Single

☐ Divorced

Section B: Knowledge on initial Neonatal Care elements for a healthy breathing neonate:

11. **Have you ever heard of Initial Neonatal Care? ***

☐ No

☐ Yes

12. **Define Initial Neonatal Care**
13. **List 5 elements of initial neonatal care:**

Timing and priority of initial neonatal care provided to a healthy neonate after birth

14 At what time after birth is initial neonatal care considered most important for the neonate?

☐ Within one hour after birth

☐ Within 24 hours

14. **Wiping liquor and meconium-stained liquor from mouth and nose while the head appears at the vulva prevent neonatal aspiration ***

☐ No

☐ Yes

15. **If the neonate is breathing well after full delivery, dry, cover with dry cloth, apply skin-to-skin contact to prevent hypothermia and early initiation of breastfeeding**

☐ No

☐ Yes

16. **What time is APGAR Score first performed**

☐ 1 and 10 minutes

☐ 1 and 5 minutes

Benefits of initial neonatal care (INC) to a healthy breathing neonate

17. Initial neonatal care practices prevent neonatal hypothermia

☐ No

☐ Yes

18. Initial neonatal care practices reduce risks of aspiration

☐ No

☐ Yes

19. Initial neonatal care evaluation Initiates neonatal resuscitation

☐ No

☐ Yes

20. Initial neonatal care practices prevent neonatal hypoglycemia

☐ No

☐ Yes

Knowledge on neonatal resuscitation **(neonates with difficulty breathing at birth)**

21. Have you heard about neonatal resuscitation?

☐ No

☐ Yes

22. .What do you understand about neonatal resuscitation?

____

____

23. If neonate is not breathing after birth due to secretion, mouth and nose should be wiped, suctioned, before stimulating

☐ No

☐ Yes

24. Ventilation should be initiated within one minute of birth to a neonate with difficulty breathing

☐ No

☐ Yes

25. During ventilation, face mask should must cover nose, mouth and chin

☐ No

☐ Yes
